# Does narrator variability facilitate incidental word learning in the classroom?

**DOI:** 10.3758/s13421-021-01228-4

**Published:** 2021-09-20

**Authors:** José Luis Tapia, Eva Rosa, Francisco Rocabado, Marta Vergara-Martínez, Manuel Perea

**Affiliations:** 1 Universidad Nebrija, Madrid, Spain; 2 Department of Psychology, Universitat de València, Blasco Ibáñez, 21, 46010 Valencia, Spain; 3 Basque Center on Cognition, Brain, and Language (BCBL), Donostia, Spain

**Keywords:** Contextual diversity, Narrator variability, Indexical information, Incidental word learning

## Abstract

Recent studies have revealed that presenting novel words across various contexts (i.e., contextual diversity) helps to consolidate the meaning of these words both in adults and children. This effect has been typically explained in terms of semantic distinctiveness (e.g., Semantic Distinctiveness Model, Jones et al., *Canadian Journal of Experimental Psychology*, *66*(2), 115, 2012). However, the relative influence of other, non-semantic, elements of the context is still unclear. In this study, we examined whether incidental learning of new words in children was facilitated when the words were uttered by several individuals rather than when they were uttered by the same individual. In the learning phase, the to-be-learned words were presented through audible fables recorded either by the same voice (low diversity) or by different voices (high diversity). Subsequently, word learning was assessed through two orthographic and semantic integration tasks. Results showed that words uttered by different voices were learned better than those uttered by the same voice. Thus, the benefits of contextual diversity in word learning extend beyond semantic differences among contexts; they also benefit from perceptual differences among contexts.

## Introduction

In an influential study, Adelman et al. (2006) found that contextual diversity, defined as the number of different documents in which a word appears, was the strongest facilitative predictor of response times (even more than word-frequency) in word-naming and lexical-decision tasks. This finding has been replicated numerous times not only in word-recognition tasks (e.g., Brysbaert et al., 2012; Cai & Brysbaert, 2010; Dimitropoulou et al., 2010; Duchon et al., 2013; Keuleers et al., 2010; Soares et al., 2015), but also during sentence reading (e.g., Chen et al., 2017; Pagán & Nation, 2019; Plummer et al., 2014). Importantly, while contextual diversity and word frequency are highly correlated (i.e., words that appear in many contexts also tend to be of high frequency), they tap onto qualitatively different cognitive and brain processes (see Vergara-Martínez et al., 2017): N400 amplitudes (i.e., an electrophysiological signature of lexical-semantic processing) are larger for low- than for high-frequency words. In contrast, N400 amplitudes are larger for high- than for low-contextual diversity words.

More important for the purposes of the present research, contextual diversity also plays a facilitative role during word learning. A correlational analysis of the CHILDES database (this database contains child-directed speech toward 12- to 60-month-old children) revealed that children learned more rapidly words that occur in many contexts (Hills et al., 2010). The relationship between contextual diversity and word learning was also examined experimentally with adult readers (Jones et al., 2012). The Jones et al. (2012) study used an artificial language-learning paradigm in which participants learned a lexicon made up of 12 new words: four subject words (unfamiliar images), four object words (geometric shapes constructed from *geons*), and four locatives (i.e., above, below). During the training phase, participants viewed slides that consisted of an image and a three-word sentence that described that image. The words belonging to the low-diversity condition always appeared in the same semantic context (i.e., eight encounters with the same sentence and the same image), whereas the words belonging to the high-diversity condition appeared in different semantic contexts (i.e., eight encounters with different sentences and their corresponding images). After the training phase, participants completed a pseudo-lexical decision task (“is the item a word in this language?”). Response times were faster for those words learned in semantically distinct contexts than for those learned in redundant contexts.

To explain the facilitative effect of contextual diversity on word learning, Jones et al. (2012) proposed the Semantic Distinctiveness Model (henceforth, SDM). A basic assumption of this model is that the memory enhancement of a word meaning does not only benefit from mere repetition, but rather from semantic changes across contexts where learning takes place. When we learn words, the maximal strength of word encoding occurs at the first encounter. In further encounters with that word, its strength codification will benefit from differences between the context in which it is re-encountered and our previous lexical portrayal. That is, each new encounter with a word contributes to learning it but, the greater the difference between the current context and the contexts in which the word has been previously experienced, the greater the benefits for word learning. Similarly, the hypothesis of contextual variability (see Bolger et al., 2008) posits that new concepts’ learning is more effective if it occurs in different sentences or contexts than if it occurs in a single context. This hypothesis argues that every encounter with a word in a distinct context creates a memory trace with the context’s episodic information, so that the more memory traces a word has, the easier it will later be retrieved (Fukkink et al., 2001; Nagy et al., 1985; Nagy et al., 1987).

Consistent with the SDM, Johns et al. (2016) found a benefit of the diversity of contexts in an experiment where university students learned new invented words by reading fragments extracted from articles, books, and newspapers. Fragments could comprise five passages either from a single discourse topic (low diversity) or from a number of distinct topics (high diversity). Results showed faster and more accurate recognition times in a lexical decision task for those words learned in high than in low distinctive passages.

In a more applied scenario, Rosa et al. (2017) examined the role of contextual diversity on incidental word learning with Grade 3 children in a 4-day experimental setting in the classroom. During the initial 3-day training phase, the students read three texts (a fable, an expository text with science contents, and a text with math problems) containing the novel words. (As is common in classroom settings, children were told that they had to guess from the context for those words they did not know.) In the high-diversity condition, novel words were presented in three semantically different contexts (e.g., math, sciences, language), whereas in the low-diversity condition they were presented in three semantically similar contexts (e.g., always in sciences contexts). On the fourth day, learning was assessed through two memory tasks (free recall and recognition) and two semantic and orthographic integration tasks (matching words with pictograms and multiple-choice task with lexical distractors). The results of this study showed that the novel words presented in semantically different contexts were learned better than those novel words presented in semantically similar contexts in all four dependent variables. In the same line, another study using the eye-movement paradigm examined how contextual diversity variations (i.e., words presented in the same sentence or in different sentences) influenced word learning in adults (Pagán & Nation, 2019). A retrieval task revealed that words presented in diverse contexts were learned better than those learned in the same context.

The results from the above-cited experimental studies have been interpreted in terms of the lexical-semantic enhancement produced by contextual diversity, within the framework of the SDM. Notwithstanding, while semantics may well underlie the effect of contextual diversity in word learning, Johns et al. (2016) acknowledged that “*this could also be seen as an episodic effect*,” the reason being that the manipulation of contextual diversity “*could be interpreted as an encoding variability manipulation (*Bower*,* 1970*), in which distinctive contexts lead to differential encoding, resulting in the observed differences in task performance*.” (p. 1219). The present experiment is an attempt to fill this gap in the literature. In particular, the focus was on assessing the impact of an encoding manipulation (talker variability) on word learning.

Language is stored in the memory in a high-dimensional manner that includes concrete and detailed patterns of non-linguistic auditory cues associated with the speech production, such as the speaker’s pitch and prosodic characteristics (e.g., speaking tempo, pronunciation, and intonation). These non-linguistic cues are better understood as indexical information, and its variability effect over the processing and representation of linguistic auditory information has been a matter of debate in the past decades (see Port, 2007, for a review). With respect to word learning, indexical information plays a relevant role in more recent theories of speech perception. For example, episodic/exemplar-based models (see Goldinger, 1998) assume that indexical information is preserved and stored in memory representations along with phonetical auditory information, which, in turn, would facilitate speech perception (see Martin et al., 1989; Sommers et al., 1994). Consistent with this view, recent research has shown that the voice of the speaker is incorporated into memory representations along with other types of co-occurrences as background sounds (Pufahl & Samuel, 2014). Across six experiments, Pufahl and Samuel (2014) showed that the recognition of a word is affected by the ambient sounds in which it has been previously experienced, in the same way that the recognition of a sound is affected by the words with which it has previously co-occurred. They concluded that the linguistic and non-linguistic aspects of the auditory input are not automatically segregated, but stored together to form the episodic traces that constitute the basic substrate of the mental lexicon. Accordingly, one might expect that contextual variations in indexical aspects like the speaker’s voice would also have a beneficial effect on incidental vocabulary learning in natural contexts. Keep in mind that one of the key factors to impact vocabulary growth is the oral language environment (Ramírez-Esparza et al., 2014). In this line, storytelling or book reading-aloud settings are among the key driving factors of lexical growth in childhood (Elley, 1989; Ninio & Bruner, 1978; Robbins & Ehri, 1994). Interestingly, compared to the direct teaching of vocabulary, the incidental learning of words accounts for the vast number of words that are learned during childhood and adolescence (see Biemiller & Slonim, 2001; Nation, 2006). Indeed, we may find pedagogical implications in the present study: as strong vocabulary skills have been linked to better reading outcomes over time (Dickinson & Tabors, 2001; Hart & Risley, 1995; Scarborough, 2005), our study may help understand the factors that aid vocabulary acquisition during incidental learning (i.e., contextual diversity).

The main goal of our study was to examine whether an encoding (non-semantic) manipulation of contextual diversity could facilitate incidental word learning in the classroom – we chose Grade 3 students to parallel previous work (see Rosa et al., 2017). In the present experiment, novel words were presented in carrier fables that were uttered either by the same or by different narrators, while keeping constant the semantic nature of the contexts in which the novel words were presented. For ecological validity, the experiment took place at the children’s school during regular hours.

Children were presented with novel words in the context of multiple fables uttered always by the same narrator or by different narrators (low- vs. high-contextual diversity). Words assigned to the low-contextual diversity condition were inserted in three fables uttered by the same narrator, while words assigned to the high-diversity condition were inserted in three fables uttered by different narrators, while keeping constant the overall frequency of each new word (see Rosa et al., 2017, for a similar strategy with a semantic manipulation of contextual diversity with Grade 3 children). To create more salient auditory contexts, the voice pitch was very different across narrators (high vs. medium vs. low pitch). The effect of contextual diversity on incidental word learning was measured in two orthographic and semantic integration tasks: (1) a multiple-choice task with lexical distractors, and (2) a picture-word matching task.

Clearly, if each voice creates a distinct context when learning new words, we expect that words uttered by different voices (high-contextual diversity words) would be better learned than those words pronounced by the same voice (low-contextual diversity words). This outcome would support the view that variability of indexical information, such as voice pitch, plays an important role during word learning (Goldinger, 1998; see Pufahl & Samuel, 2014, for episodic accounts of word learning). In contrast, a null effect of (auditory) contextual diversity in the present scenario would suggest that the contextual diversity effect on the learning of new vocabulary is primarily semantic in nature (see Jones et al., 2012).

## Materials and methods

### Participants

Ninety-two third grade children from a subsidized private school in Valencia, Spain, participated in the study. The average age was 8 years (range: 8–9 years). All participants (children’s parents) provided written informed consent before participation in the experiment. The experimental procedures were approved by the Experimental Research Ethics Committee of the University of Valencia. Thirty-nine of the initial 92 participants were excluded from the final sample due to several reasons: 12 of them showed previous learning difficulties (attention deficit disorder, learning disability, and autism spectrum disorder), and 27 missed some of the experimental sessions. Of the remaining 53 participants, 29 were boys.

### Materials

The words to be learned, referred to as experimental words, were the same as in the Rosa et al. (2017) experiment: 12 words in Spanish (average length: 7.5 letters, range: 6–11), of which 11 were nouns and one was an adjective. These words did not occur in the LEXIN primary school lexical database in Spanish (Corral et al., 2009) and had a very low frequency of use (mean = 0.15, range 0–0.9) in the EsPal Spanish subtitle database (the average was less than 0.2 per million) (Duchon et al., 2013). We also checked that none of these words were known by children of this age – this was verified by presenting these words to a different sample of 53 children.

From these experimental words, we created two counterbalanced sets of materials so that each word appeared in the high- and the low-diversity condition. Each set was composed of a total of nine short fables. The fables were equal in length (155 words each) and readability indexes (e.g., Flesch-Szigriszt [IFZS] readability index, Barrio, 2015) ranged between normal and very easy (Median = 72). The teachers in charge of the children at test assessed that the fables were appropriate to their grade level.

In all the texts, we provided semantic clues, next to each experimental word, so that the participants could infer their meaning, without providing an explicit description. The texts were manipulated to be similarly informative about experimental word meaning, i.e., the number of semantic clues remained constant in all texts for each of the experimental words. Appendix A shows an example of the three complete texts in which the word “forage” was inserted.

Every text included four different experimental words (while keeping gender, number, and meaning stable) and were recorded in audio by three different voices (high, medium, and low voice pitch).^1^ The recordings were edited with Audacity software (version 2.3.0; Audacity Team, 2018) and had the same volume and similar durations (between 58 and 66 s). Assignment to the perceptual variability was fully counterbalanced by items. This is, in Set A, words 1–6 belonged to the high-diversity condition (i.e., three different voices), while words 7–12 belonged to the low-diversity condition (i.e., always the same voice). The opposite distribution was used to create Set 2 (i.e., words 1–6 belonged to the low-diversity condition and words 7–12 belonged to the high-diversity condition). Perceptual variability was manipulated within-subject so that all participants listened to half of the fables by one narrator and the other half by three different narrators. The distribution of the experimental words (and their equivalents in English) in set A and B are presented in Appendix B and Appendix C, respectively. To guarantee that the participants paid attention to the audios, participants were presented with two multiple-choice comprehension questions after each fable.

Open practice statement: The experimental materials and data are openly available at https://osf.io/2uxjw/?view_only=aa23cf9fdca54e7aa958ceab33b75215.

### Procedure

Both the training and the evaluation phase were carried out in groups in the school’s computer classroom during the regular school hours. Before starting the training phase, all participants were randomly assigned to one of the two experimental sets. Participants were seated individually on each computer, and they received task instructions from the experimenter as well as visually on the screen.

To maximize the learning process, the training phase was distributed over three consecutive days. In each of these 3 days, the students listened to three fables that were read by a high, medium, or low voice pitch narrator. As shown in Appendix B and Appendix C, each fable carried four experimental words. In order to control the overall frequency of each new word, every experimental set was presented three times in its corresponding experimental condition (high or low contextual diversity), depending on the counterbalance set. This strategy was based on a previous study by Rosa et al. (2017) in which contextual diversity effects were obtained with only three repetitions of the experimental words. In that experiment the texts were visually exposed, and the contextual diversity was semantically manipulated. After listening to each fable, students had to respond to two three-alternative forced-choice comprehension questions, which were presented in both visual and auditory modalities.

The assessment session took place on the fourth day. We employed two tasks: a multiple-choice task and a picture-word matching task. We employed these two tasks to assess the recall and recognition of new words’ semantic and word-form integration. On the one hand, the multiple-choice task mainly assessed recognition memory of both aspects. Participants had to read a sentence and predict the last word (novel-word), which was missing, among four alternatives (the correct word, two orthographic distractors, and a phonological distractor). The inclusion of these distractors allowed measurement of whether the form of the novel-word had been well established. On the other hand, the picture-word matching task required both recall and recognition of the novel-words’ meaning, as the words were not presented in the written modality. These tasks were designed and registered using OpenSesame (Version 3.2.8; Mathôt et al., 2012). The order of assessment was the same for high-contextual and low-contextual diversity conditions. The multiple-choice task was run first, followed by the picture-word matching task – there was a 5-min break between them.

#### Multiple-choice task

This task was equivalent to that used by Rosa et al. (2017) and comprised 12 incomplete sentences presented visually on display where the last word was missing. Each sentence was followed by four-alternative forced-choice options, only one of which (the experimental word) was adequate to finish the sentence. The foils were constructed so that two of them differed only by one letter from the experimental word, and the third was orthographically different but phonologically similar to the experimental word. The presentation order of the items was randomized, and the participants were aimed to click on the correct answer with no time limit. This evaluation task is presented in Appendix D.^2^

#### Picture-word matching task

In this task, a 4 × 4 matrix composed of 16 images (which represented the referents of the 12 experimental words plus four fillers) was presented on the computer screen along with the audio of one experimental word. Participants were required to select the image that matched the audio. Each of the 12 trials started with the presentation of a fixation signal (+) for 500 ms, immediately followed by the image matrix and the recorded audio of one experimental word. All the images remained on the screen until the participant’s response. Participants had one attempt for each stimulus –with no time limit – and there was no feedback after response. The presentation order of the trials was randomized. An example screen display is illustrated in Appendix E.

### Analysis and results

The overall average percentage of correct answers for the three-choice comprehension questions after each fable across the three training days was 76% (33.3% would be chance), thus indicating that participants listened mindfully to the fables. Participants’ comprehension scores were similar across the 3 days (74.2, 76.7, and 76.4%, respectively, p = .771). Table 1 shows the descriptive statistics (mean and standard deviation) for each of the tasks. For the inferential analyses, we employed generalized linear mixed-effects models in R (R Core Team, 2021) using lme4 (Bates et al., 2015). The fixed factor was Contextual Diversity (high diversity vs. low diversity). For each dependent variable, we employed the maximal random effect structure model that successfully converged – these were Dependent_Variable ~ CD + (1 | subject) + (1 | item). As the dependent variable was binary (1 = correct; 0 = incorrect), it was modeled with the binomial distribution (i.e., *family = binomial*).

**Table 1:** Descriptive statistics for each of the dependent variables in the experiment (mean hit ratio with standard errors in parentheses)

		Picture-word matching task	Multiple-choice task
Contextual Diversity	High	0.211 (0.22)	0.547 (0.25)
	Low	0.160 (0.16)	0.434 (0.23)

#### Picture-word matching task

Accuracy rates were significantly higher for the novel words when uttered by several narrators than when uttered by only one narrator, *b* = -0.5023, *SE* = 0.1725, *z* = -2.912, 95% CI (-0.844, -0.165), *p* = 0.0036; for the interested reader, the parallel ANOVA also showed a facilitative effect of contextual diversity, *F*1(1,51) = 4.19, *η*^2^ = .076, *p* = .046.^3^

#### Multiple-choice task

This task revealed higher accuracy for the novel words when uttered by several narrators than when uttered by only one narrator (see Table 1), although the effect was slightly below the traditional criterion for significance, *b* = -0.4173, *SE* = 0.2172, *z* = -1.921, 95% CI (-0.862, 0.0161), *p* = 0.0547; note that the ANOVA also revealed an effect of contextual diversity, *F*1(1,51) = 10.82, *η*^2^ = 0.175, *p* = .002.

## Discussion

In the present experiment, we investigated whether contextual diversity – operationalized in terms of narrator diversity – had a facilitative effect in the incidental acquisition of novel words by Grade 3 children (around 8 years old) in the classroom. Results from the two tasks (i.e., multiple-choice and picture-word matching) showed that novel words presented in fables uttered by different narrators (high-contextual diversity) were learned better than novel words presented in fables uttered by the same narrator (low-contextual diversity).

Our findings highlight the beneficial effect of contextual diversity in word learning, in line with prior experiments (Jones et al., 2012, Johns et al., 2016; Pagán & Nation, 2019; Rosa et al., 2017). The above-cited studies addressed the remarkable impact of contextual diversity in the consolidation of word learning in terms of semantic variability. Those findings were captured by the SDM model, a distributional model of semantic memory where the encoding strength for a word in a given context depends on the semantic content overlap between the current context and the representation of that word in memory: the larger the overlap, the weaker the word is encoded. Ours is the first study to address whether the impact of contextual diversity on incidental word learning extends beyond semantics by manipulating diversity at a perceptual rather than at a semantic level. Specifically, we manipulated the perceptual variability (the number of different narrators), whereas the semantic context in which words appeared was preserved across the high- and low-contextual diversity manipulation. The advantage of perceptual variability in word learning, as obtained in the present experiment, suggests that variating the perceptual features of the learning context alone also has an impact on incidental word learning, thus reinforcing the importance of the codification and storage of indexical aspects on the formation of lexical representations (Goldinger, 1998; Goldinger et al., 1991).^4^

Therefore, while semantics undoubtedly play a fundamental role in the effect of contextual diversity during word acquisition – as shown by Johns et al. (2016) – other modulating components such as perceptual distinctiveness also need to be incorporated in any theoretical model that aims to explain the mechanisms underlying the learning of new words. Specifically, our findings provide empirical support to episodic theories that suggest that auditory-perceptual details are stored in memory during word learning (e.g., Goldinger, 1998), as well as those models that treat lexical representations as a proper subset of auditory memory representations (Pufahl & Samuel, 2014). In this line, note that the retrieval of a specific element is more likely when it is involved in a rich network than when it is encoded in isolation (Anderson & Bower, 1972; Craik & Tulving, 1975; Tulving & Thomson, 1973). Accordingly, changes in the perceptual instantiation of the same stimulus (i.e., different encounters of the same word) may lead to a richer episodic representation which, in turn, increases the likelihood of retrieving information about the experienced stimuli in memory. Indeed, memory researchers have long acknowledged that it is not the number of repeated exposures to an item that affects retrieval, but rather the distinction of these exposures in time and context (e.g., see Glenberg, 1976, 1979). Under the premise that perceptual distinctiveness creates a new context, the more contexts in which an item occurs, the more likely it is that the item will be needed in a new context (i.e., “*principle of likely need*”; see Anderson & Milson, 1989). Additionally, and according to the episodic based models, our findings indicate that words can be incidentally learned (spelling, semantics, and phonology) through reading, and that this learning is more effective if there are contextual variations between the different experiences (episodes) with the word (Nelson et al., 2005). However, the input model may modulate the strength and quality of the memory traces (Perfetti, 1997). Indeed, visual information may entail more accessible memory traces than auditory information (Dean et al., 1988; Gallo et al., 2001). This fact could explain the low hit rate in our experiment compared with that of Rosa et al. (2017), in which the words were presented visually during the training phase.

As pointed out by one reviewer, the facilitative findings of contextual diversity reported here might appear at odds with previous findings from Johns et al. (2016), where the same novel words that showed a processing advantage in recognition tasks (via memory strength in the SDM) also showed a processing disadvantage in semantic judgment tasks (via similarity vectors). This last finding was assessed by a semantic similarity judgment task (e.g., novel words vs. associates of the target meaning), showing that words learned in semantically redundant contexts led to more discriminable representations (i.e., closer to their target meaning) than those learned in semantically diverse contexts. Johns et al. (2016) concluded that recently acquired meanings were easier to disambiguate when learned across semantically similar than semantically diverse contexts. While it would have been informative to have collected semantic similarity of the novel words in the present study, we did not include this task in the design because we had no a priori predictions on why the discriminability of semantic representations would be shaped by perceptual diversity alone. Instead, we chose other tasks (e.g., multiple-choice task, picture-matching task) that reflect recognition and recollection aspects of word learning. As such, these two tasks may be interpreted as reflecting the component of memory strength in the SDM – note that, for this component, the model predicts a facilitative effect of multiple contexts for novel words.

Distributional models of semantic representations, such as the SDM, posit a formal cognitive mechanism to learn semantics from repeated episodic experience in a linguistic context (see Jones et al., 2015, for review). In this framework, multiple presentations of a word are effective as long as there is a subtle change in the context. While the SDM focuses on the semantic variability across contexts in order to explain the benefits of contextual diversity on meaning acquisition (via distributional changes in semantics that allow inference of the word’s meaning), our findings showed that the changes in context other than semantics also play a role in word learning. In fact, subtle changes in aspects of context that are unrelated to meaning disambiguation (e.g., perceptual factors) seem enough to aid the consolidation of a new word’s meaning. Therefore, distributional models of semantic representations should embrace extended notions of “context.” In other words, the cognitive mechanisms that are posed to learn semantics from repeated episodic experiences might not only consider linguistic factors (e.g., semantics), but also indexical factors such as acoustic variability.

What are the implications of the present results from a pedagogical perspective? Here we have addressed one of the factors that aid vocabulary acquisition in incidental learning (i.e., contextual diversity). The metanalysis by Wasik et al. (2016) revealed that direct explicit vocabulary instruction proved to be more efficient in the learning of new words compared to incidental instruction of new vocabulary. However, this contrasts with other reviews that have taken into account the efficiency of instruction (number of words learned divided by instructional time), along with the feasibility and worth of providing direct vocabulary instruction, which revealed that simply listening to a story was more efficient for vocabulary acquisition than reading plus extended instruction (McQuillan, 2019a, 2019b; see also Mason et al., 2008; Nagy et al., 1985). While our study was not designed to assert which of the two approaches is more effective in vocabulary instruction (see Wright & Cervetti, 2017, for a recent review), what is clear from our experiment is that incidental learning of vocabulary may benefit from a (perceptual) contextual diversity manipulation in the classroom. Specifically, we showed that the learning of novel words when uttered by several narrators was more efficient than when uttered by a single narrator in a school class scenario, a result that can serve as the basis for better tools to improve vocabulary learning. These findings might inform teachers’ and interventionists’ decisions on how to optimize vocabulary acquisition during classroom context while dealing with time efficiency. Therefore, teachers might introduce pedagogical innovations in the learning of unknown terms by exploiting the benefits of contextual diversity (i.e., changing the way that new words are introduced in the classroom). These may include distributing novel words across different teachers for the same student audience and using cross-curricular learning. We also believe that, besides its theoretical implications, the present experiment may also have practical applications in different populations and learning environments. For example, in line with the Highly Variable Phonetic Training (HVPT), auditory variability can be used in speech therapy sessions to benefit children with difficulties in the perception and pronunciation of words (Plante et al., 2014). Likewise, auditory diversity can also optimize the consolidation of semantic and phonological elements of second language learning (see Barriuso & Hayes-Harb, 2018; Frances et al., 2020, for recent evidence).

In sum, we have demonstrated that the advantages of contextual diversity on incidental word learning do not rest exclusively on semantic differences among contexts where words are encountered, but also on perceptual differences among contexts (indexical information), contributing to long-term episodic memory consolidation. To better understand the role of perceptual aspects in the contextual diversity effect, future studies should evaluate the efficacy of incidental vocabulary learning by manipulating perceptual diversity in different ways.

## Appendix A

*Example in Spanish and English of three texts (low, medium, and high pitch) in which the target word forraje (the Spanish for forage) was inserted, and the corresponding reading comprehension questions.*

## Appendix B

**Table 2:** Distribution of experimental words in the fables of Experimental set A

Medium pitch 1	Medium pitch 2	Medium pitch 3
Bermejo	Dehesa	Venado
Batracios	Raigón	Caninos
Promontorio	Promontorio	Promontorio
Guijarros	Guijarros	Guijarros
**High pitch 1**	**High pitch 2**	**High pitch 3**
Venado	Bermejo	Dehesa
Caninos	Batracios	Raigón
Vulpeja	Vulpeja	Vulpeja
Simientes	Simientes	Simientes
**Low pitch 1**	**Low pitch 2**	**Low pitch 3**
Dehesa	Venado	Bermejo
Raigón	Caninos	Batracios
Valvas	Valvas	Valvas
Forraje	Forraje	Forraje

*Note:* Target words belonging to the high-contextual diversity condition: Bermejo (Russet), Batracios (Batrachians), Dehesa (Meadow), Raigón (Stump), Venado (Venison) and Caninos (Canines). Experimental words belonging to the low-contextual diversity condition: Promontorio (Promontory), Vulpeja (Vulpine), Valvas (Valves), Guijarros (Cobblestone), Simientes (Seeds) and Forraje (Forage)

## Appendix C

**Table 3:** Distribution of target words in the fables of Experimental set B

**Medium pitch 1**	**Medium pitch 2**	**Medium pitch 3**
Promontorio	Valvas	Simientes
Vulpeja	Guijarros	Forraje
Bermejo	Bermejo	Bermejo
Raigón	Raigón	Raigón
**High pitch 1**	**High pitch 2**	**High pitch 3**
Simientes	Promontorio	Valvas
Forraje	Vulpeja	Guijarros
Batracios	Batracios	Batracios
Venado	Venado	Venado
**Low pitch 1**	**Low pitch 2**	**Low pitch 3**
Valvas	Simientes	Promontorio
Guijarros	Forraje	Vulpeja
Dehesa	Dehesa	Dehesa
Caninos	Caninos	Caninos

*Note:* Experimental words belonging to the high-contextual diversity condition: Promontorio (Promontory), Vulpeja (Vulpine), Valvas (Valves), Guijarros (Cobblestone), Simientes (Seeds) and Forraje (Forage). Experimental words belonging to the low-contextual diversity condition: Bermejo (Russet), Batracios (Batrachians), Dehesa (Meadow), Raigón (Stump), Venado (Venison) and Caninos (Canines).

## Appendix D

*Sentences presented in the multiple-choice task and their English translation.*

## Appendix E

*Screen display for the Picture-word matching task.*

## Footnotes

1 In response to one suggestion from a Reviewer, we did not include pitch as a quantitative predictor because our hypothesis was not aimed at verifying whether pitch produced differences in terms of word learning. We acknowledge that this observation opens the door for future research on whether the narrator’s pitch affects learning novel words.

2 We decided to present this test in written modality since it would have been difficult to remember the three options if they had been presented in an aural way. The change of modality with respect to the exposure phase should not be a confounding factor given that Spanish has a transparent sound-spelling system, and that 8- year-s old children have already learned sound-spelling correspondences. Notwithstanding, as suggested by a rReviewer, the asymmetry on information transfer from visual-to-auditory rather than from auditory-to-visual may partly explain the low hit rates at test in the present study. This is something to take into account in further research.

3 List was included in the ANOVAs to reduce the error variance due to the counterbalanced lists (Pollatsek & Well, 1995)

4 In the present experiment, we did not assess the role of the children’s reading ability. We acknowledge that future studies should also consider whether this factor modulates contextual diversity benefits. 
